# Comprehensive overview of how to fade into succinate dehydrogenase dysregulation in cancer cells by naringenin-loaded chitosan nanoparticles

**DOI:** 10.1186/s12263-024-00740-x

**Published:** 2024-05-27

**Authors:** Eman M. Ragab, Abeer A. Khamis, Doaa M. El Gamal, Tarek M. Mohamed

**Affiliations:** Biochemistry Division, Chemistry Department, Faculty of Science, Tanta University, Tanta, Egypt

**Keywords:** Mitochondria, Cancer, Succinate dehydrogenase, Epigenetics, Chitosan, Naringenin

## Abstract

Mitochondrial respiration complexes play a crucial function. As a result, dysfunction or change is intimately associated with many different diseases, among them cancer. The epigenetic, evolutionary, and metabolic effects of mitochondrial complex IΙ are the primary concerns of our review. Provides novel insight into the vital role of naringenin (NAR) as an intriguing flavonoid phytochemical in cancer treatment. NAR is a significant phytochemical that is a member of the flavanone group of polyphenols and is mostly present in citrus fruits, such as grapefruits, as well as other fruits and vegetables, like tomatoes and cherries, as well as foods produced from medicinal herbs. The evidence that is now available indicates that NAR, an herbal remedy, has significant pharmacological qualities and anti-cancer effects. Through a variety of mechanisms, including the induction of apoptosis, cell cycle arrest, restriction of angiogenesis, and modulation of several signaling pathways, NAR prevents the growth of cancer. However, the hydrophobic and crystalline structure of NAR is primarily responsible for its instability, limited oral bioavailability, and water solubility. Furthermore, there is no targeting and a high rate of breakdown in an acidic environment. These shortcomings are barriers to its efficient medical application. Improvement targeting NAR to mitochondrial complex ΙΙ by loading it on chitosan nanoparticles is a promising strategy.

## Introduction

Cancer is a serious global public health issue. It is the second greatest cause of mortality, therefore advanced-stage disease and mortality rates may rise because of delayed diagnosis and treatment [74]. The American Cancer Society estimates the number of new cancer cases and deaths annually and compiles the most recent data on population-based cancer occurrence and outcomes. It is anticipated that there will be 609,820 cancer-related deaths and 1,958,310 new cancer cases in 2023 [55]. Recently cancer has been recognized as a metabolic disease. Cancer cells find many metabolic pathways to meet their needs. Among these OXPHOS plays a substantial role in the development of many cancer cells. OXPHOS supplies enough energy for the survival of tumor tissue while also regulating the conditions that promote tumor growth, invasion, and metastasis [11, 41, 60].

Targeting tumor bioenergetics may be a promising approach to cancer therapy among the various new techniques, as it involves rewiring. The biochemical pathways linked to energy metabolism play a role in the development, persistence, and resistance of tumors to treatment. As a result, addressing mitochondrial metabolism, namely redox metabolism, is a promising therapeutic strategy. In this review, we discuss recent findings on modifications to the primary metabolic pathway that promotes cancer growth, such as endogenous succinate dehydrogenase SDH inhibitors that lead to succinate accumulation or dysregulation of (SDH). We additionally talk about the impact of succinate on angiogenesis, cell invasion, and migration, as well as how it triggers metabolic and epigenetic modifications that play a role in the development of cancer.

## Respiratory chain and mitochondrial energy metabolism

**Fig. 1:**
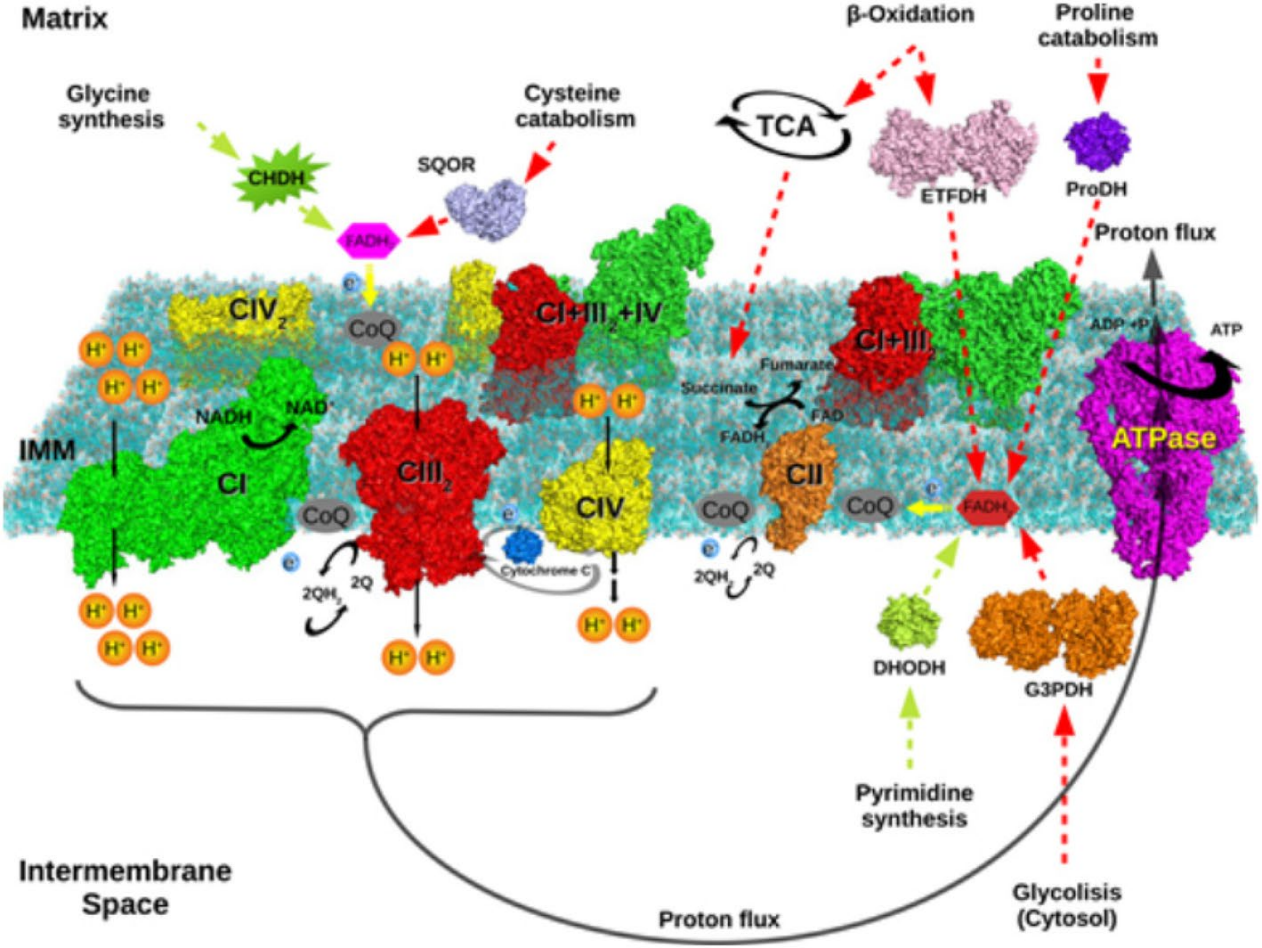
The respiratory chain and mitochondrial energy metabolism. The end product of lipid and carbohydrate metabolism is acetyl-coenzyme A (Acetyl-CoA), which is oxidized to create CO_2_ by Krebs cycle processes. These reactions produce high-energy electrons (e^−^), which eventually enter the respiratory chain and decrease molecular oxygen (O_2_) to make water (H_2_O). This process releases energy, which is then used to create the electrochemical gradient that allows complex V to synthesize ATP by pumping protons (H^+^) across the inner membrane of the mitochondria [12]

Mitochondria are organelles well-known for their role in cellular respiration, particularly in linking the production of ATP and the reduction of molecular oxygen (O_2_). Numerous important metabolic pathways, including the urea cycle, oxidation, citric acid cycle (CAC), and heme synthesis, are housed in mitochondria [28]. The TCA cycle’s main role is to completely oxidize acetyl-CoA to CO_2_. Because it is necessary for the synthesis of the high-transfer-potential electron carriers flavin adenine dinucleotide (FADH_2_) and nicotinamide adenine dinucleotide (NADH H^+^), it is recognized as the main process in energy metabolism [78]. The electron transport chain (ETC) is a network of electron carriers that allows high-energy electrons to go from NADH H^+^ and FADH_2_ to the terminal electron acceptor O_2_ as described in (Fig. 1). Mainly, the ETC does its function of establishing an electrochemical gradient across the inner membrane of the mitochondria (IMM), which is necessary for chemiosmotic ATP synthesis (OXPHOS) [20]. The respiratory chain complexes are among the largest membrane protein complexes in the cell. They transfer electrons from NADH and succinate to molecular oxygen via several redox cofactors including flavins, iron-sulfur clusters, hemes, and ions. The proton pumps are NADH: ubiquinone oxidoreductase or complex I, which oxidizes NADH, transfers two electrons to ubiquinone and translocates four protons across the membrane, ubiquinol: cytochrome c oxidoreductase (complex III), which transfers electrons from ubiquinone to the peripheral electron carrier cytochrome c while translocating 4 protons, and cytochrome c oxidase (complex IV), which transfers electrons from cytochrome c to molecular oxygen and translocate two protons. In total 10 protons per NADH molecule are translocated across the IMM [12, 19].

## Succinate-ubiquinone oxidoreductase (succinate dehydrogenase (SDH))

**Fig. 2:**
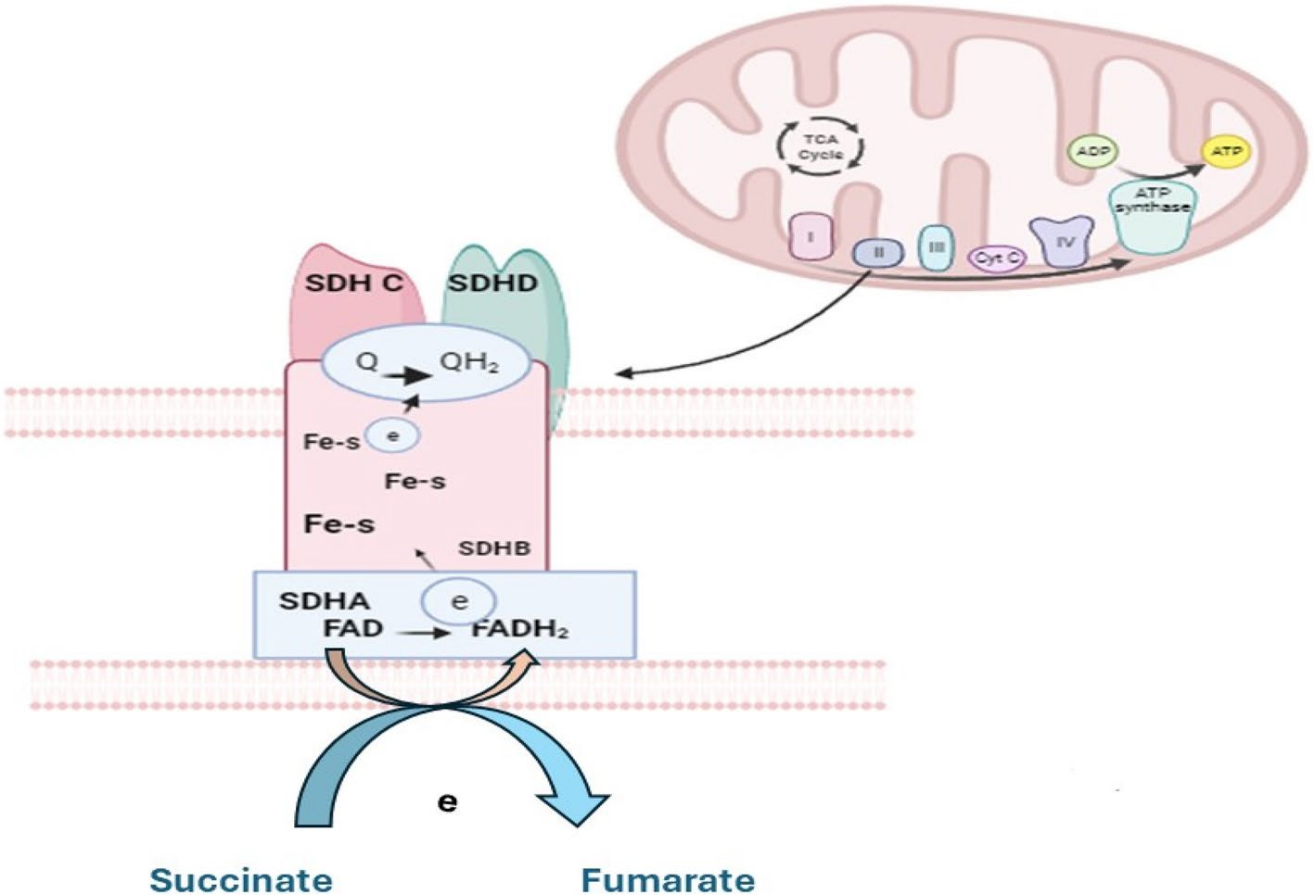
The catalytic component SDH subunit A, which carries the flavin cofactor (FAD) to transfer electrons from succinate to the Fe-S center in the SDH subunit B subunit, then the electrons are subsequently transported to complex III by the ubiquinone pool that is integrated into the SDHC and SDHD subunits [43]

SDH is a complex II mitochondrial enzyme that plays an active role in the electron transport chain within the mitochondria as well as the TCA cycle. SDH catalyzes the conversion of succinate to fumarate in the TCA cycle and carries electrons to the respiratory chain's ubiquinone (coenzyme Q) pool. The catalytic subunits in the hydrophilic head of SDH that extend into the mitochondrial matrix are subunits A (SDHA) and B (SDHB) [44]. These activities are carried out by the membrane-anchoring and ubiquinone-binding SDH subunits, SDHC, and SDHD, respectively as illustrated in (Fig. 2). The SDH assembly factor (SDHAF) is required for the flavination of SDHA, which is required for the formation of the SDH complex [67]. The 16 exons of the SDHA gene are located on chromosome 5p15.33 [10, 22]. The succinate binding site is a component of SDHA, which is the main catalytic element that converts succinate to fumarate. The SDHB gene, located on chromosome 1p35-36.1, consists of 8 exons [2, 80]. It facilitates electron transport to the ubiquinone pool and has three Fe-S centers. The location of the SDHC gene is 1q21, and it has 6 exons [36, 67], and the four exons of the SDHD gene are located on chromosome 11q23 [4, 52]. SDHC and SDHD bind to ubiquinone to generate protons, which subsequently convert to ATP [14].

### mRNA expression regulation

**Fig. 3:**
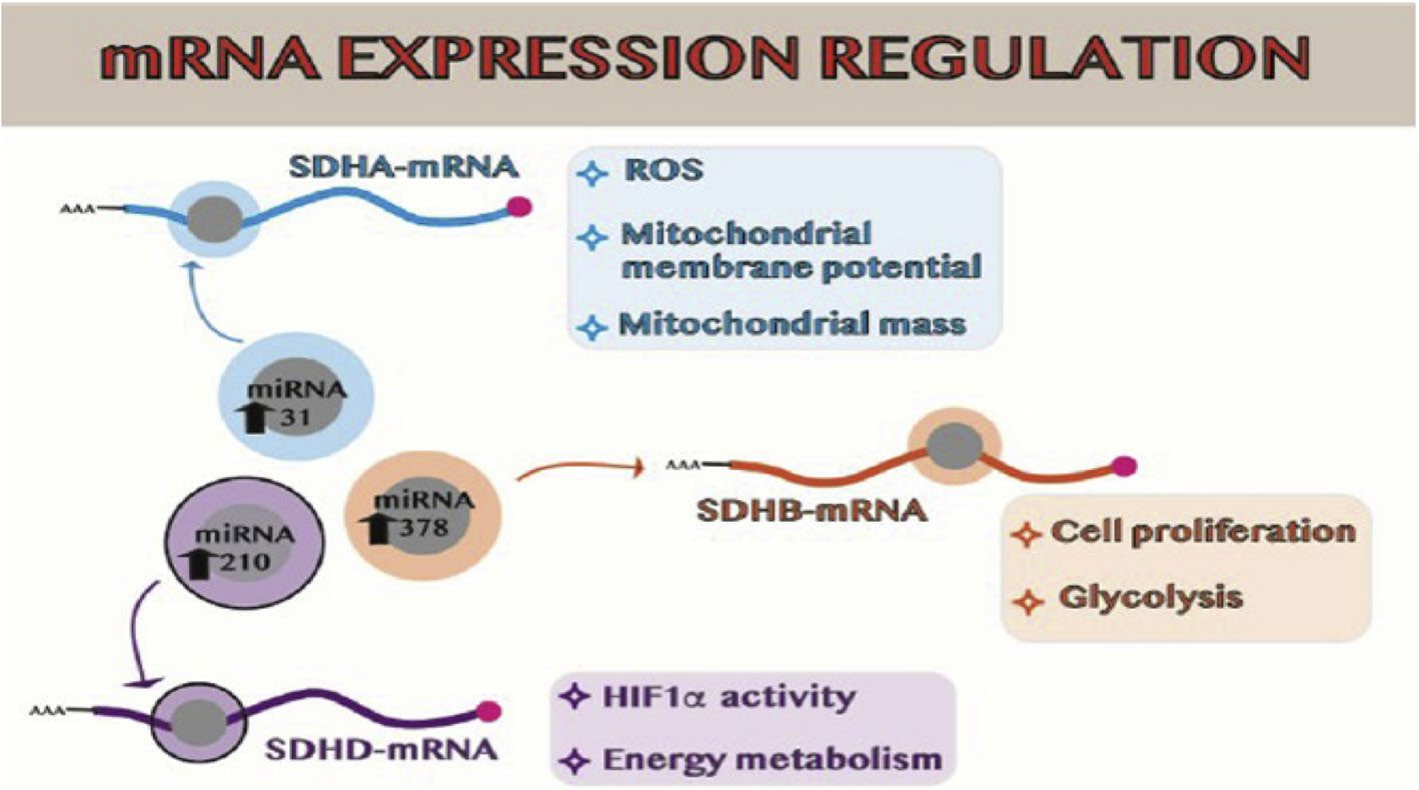
Schematic representation of SDH expression and activity alterations: SDH mRNA targeting miRNAs [14]

In addition to the SDH gene’s mutational status, other factors that affect SDH deficiency include methylation of the gene’s promoter and the expression of miRNAs. A full disassembly of the SDH complex and the ensuing lack of enzymatic activity is determined by the hypermethylation of the promoter of the SDHC subunit, which has been demonstrated to result in decreased mRNA expression of the subunit. Specifically, miRNAs -210, -31, and -378 are the three miRNAs that have been discovered to be overexpressed in cancer cells following radiation therapy. They target SDH mRNA. With significant effects on HIF1 activity and cell metabolism, miRNA-210 targets SDHD mRNA; miRNA-31 targets SDHA mRNA, which modifies ROS generation, mitochondrial membrane potential, and mitochondrial mass; and miRNA-378 targets SDHB mRNA, which encourages cell proliferation and a metabolic shift away from oxidative metabolism as demonstrated in (Fig. 3) [16, 46].

### Post-translation modification

#### Phosphorylation

SDH activity is governed at the post-translational stage by several methods, notably phosphorylation. For instance, the Y215 of the SDHA needs to be phosphorylated by c-Src kinase for the electron transfer of complex II to be successful [37, 50, 79]. It's also been found that in certain conditions like oxidative stress, SDHA phosphorylation is elevated, leading to improved respiration dependent on Complex II. Moreover, studies have shown that the dephosphorylation process, assisted by PTEN-like mitochondrial phosphatase-1 (PTPMT1), correlates to adjusting glucose levels and contributes to the disruption of SDH activity [15, 32, 40].

#### Deacetylase enzymes (SIRT3)

The acetylation of thirteen lysines in SDHA has been demonstrated, and the absence of SIRT3, a NAD-dependent deacetylase, has been shown to decrease the activity of the enzyme [68, 72, 82]. However, further research is required to understand the interaction between SDH and SIRT3 and how cells adapt to metabolic changes. SDH dysfunction, whether due to loss, reduction, or dysregulation, leads to altered metabolic traits through succinate accumulation [14, 33]. Naringenin, an AMPK-SIRT3 activator, has been found to protect against mitochondrial oxidative stress and maintain mitochondrial function during ischemia–reperfusion injury. Naringenin shows potential as a therapeutic option for mitochondrial dysfunction associated with various disorders [77, 82].

### Role of TRAP1 on succinate dehydrogenase activity

Tumor necrosis factor receptor-associated protein 1 (TRAP1) plays a vital role in different tumor types and is a significant member of the mitochondrial heat shock protein 90 (Hsp90) family. TRAP1, also called Hsp75, is the mitochondrial counterpart of the molecular chaperone Hsp90 and exhibits a remarkable similarity in terms of amino acid sequence and domain structure. It is involved in various biological processes within the mitochondria [3, 21]. Members of the Hsp90 family are molecular chaperones that play a crucial role in the breakdown of proteins following aggregation, unfolding, or misfolding. These chaperones facilitate conformational modifications in proteins, enabling them to attain specific subcellular positions or form complex structures [38, 62]. The biochemical outcomes of these processes involve the integration of signaling and metabolic pathways, resulting in a comprehensive proteostasis that can be viewed as a dynamic mechanism for ensuring the quality control and efficiency of the proteome in response to varying environmental circumstances [21, 79]. TRAP1’s presence in the mitochondria makes it a suitable choice for regulating metabolic processes occurring within these organelles and responding to harmful conditions that affect mitochondrial function. Several studies have indicated that TRAP1 expression or activity tends to rise in response to pathological circumstances [73, 79].

Furthermore, alterations in TRAP1 have been detected in a situation of sudden onset inflammation, which was also correlated with an imbalance in cellular redox status [59]. In a pediatric patient diagnosed with Leigh syndrome, a mitochondrial disorder, there is an observed correlation with gastrointestinal dysmotility, chronic fatigue, and persistent discomfort. Additionally, a small subgroup of individuals with complex developmental syndromes, characterized by kidney malformations and abnormalities in other body regions, also exhibit this connection [9, 48, 56, 79]. Hence, it has been proposed that TRAP1 safeguards the mitochondria against damage in models of renal fibrosis [7], and a proband with thyroid and kidney malignancies was found to have a TRAP1 mutation [35]. Cancer model studies provide the main evidence suggesting that the chaperone may play a detrimental role. This is because TRAP1 is found to be significantly expressed in groups of patients with hepatocellular carcinoma (HCC**)** [31, 79]. Small cell lung cancer high-grade glioma, breast cancer [61], ovarian, kidney, prostate, esophageal, and colorectal cancer are diseases in which a connection has been established with advanced stage, metastasis, and an unfavorable prognosis [5]. The upregulation of TRAP1 occurs before the development of cancerous changes and is observed only in lesions that progress to carcinoma in colorectal cancer associated with ulcerative colitis and in a specific animal model of hepatocellular carcinoma (HCC) progression. Although elevated TRAP1 levels are associated with a negative prognosis, strong TRAP1 expression is correlated with a higher chance of lymph node metastasis. However, the exact pathological significance of TRAP1 in cancer remains unclear [71]. These results indicate that TRAP1 plays an important part in the metabolic adaptations that facilitate the growth of tumors. As a result, researchers have conducted studies to gain a better understanding of its biochemical functions in different cancer scenarios. In recent years, there have been advancements in comprehending TRAP1’s structure, mechanism of action, interaction partners, and the effects it has on cellular functions. Additionally, the discovery of specific inhibitors has contributed to our understanding of TRAP1 as a key regulator in tumor energy metabolism.

**Fig. 4:**
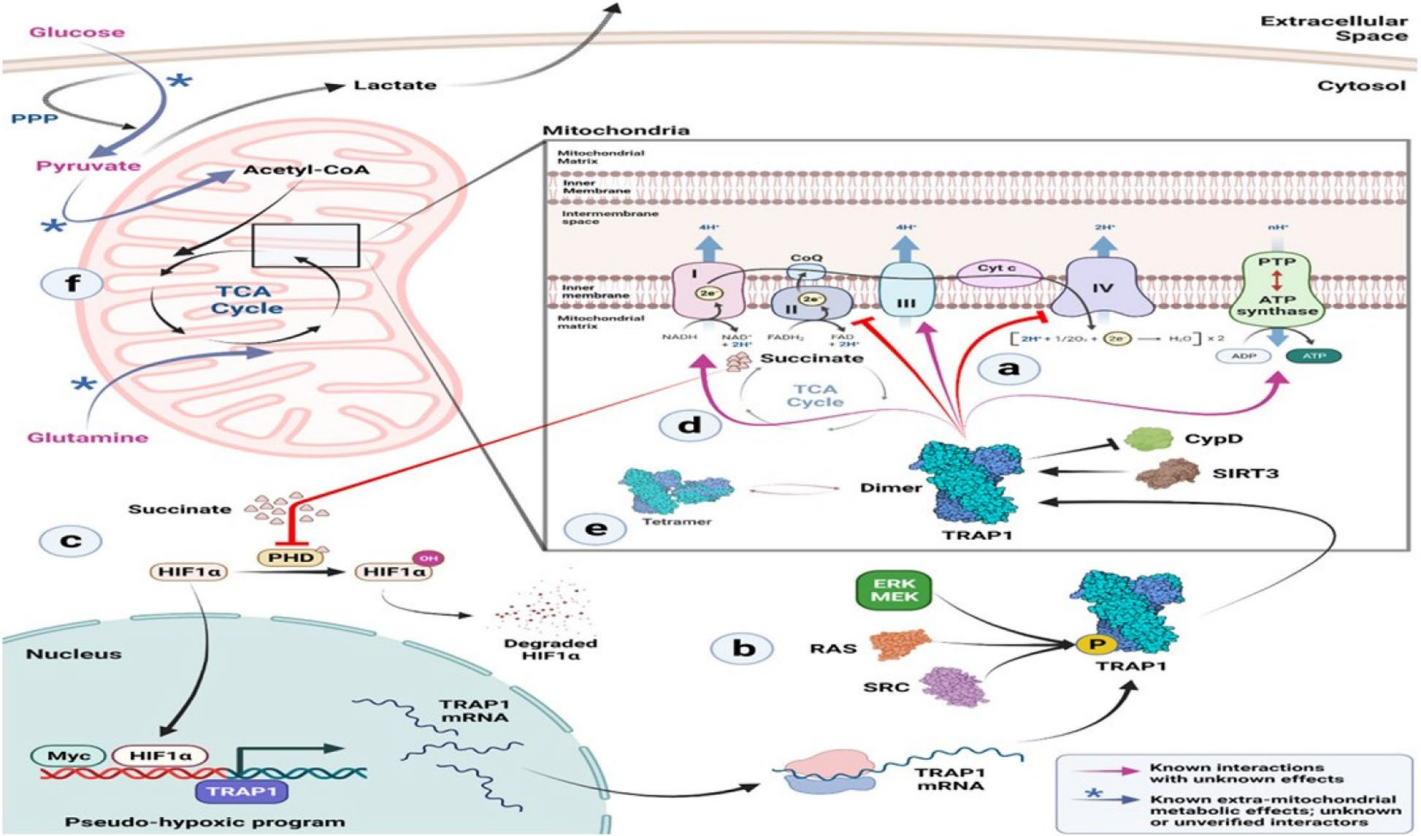
The biological process via which TRAP1 affects SDH. **a** The electron transport chain (ETC) complexes II and IV are bound by TRAP1 in the mitochondria, which stops them from functioning. TRAP1 is known to suppress cyclophilin D (CypD), which stops the permeability transition pore (PTP) from opening and the consequent activation of apoptosis by the release of cytochrome c. This is due to TRAP1's interaction with the protein deacetylase SIRT3. **b** Additionally, TRAP1 becomes more active when it is phosphorylated through a variety of pathways. Note that it is currently unclear if this happens before or after TRAP1 is imported into the mitochondria. **c** Because TRAP1 suppresses the ETC complex II, succinate accumulates as a result. This inhibits prolyl hydroxylases’ ability to stabilize HIF1 in the cytosol. Stabilized HIF1 and Myc initiate a pseudo-hypoxic program, which increases TRAP1 gene expression even more. **d** Inside mitochondria, TRAP1 interacts with ETC complexes I, III, and V (ATP synthase), but the implications of this interaction remain unknown. **e** TRAP1 dimers and tetramers coexist in the mitochondrial matrix, but it is yet unclear what influences the ratio of dimer to tetramer and whether or not they are functionally significant. **f** The carbon preference of mitochondria is influenced by TRAP1 levels. In TRAP1 KO cells, the carbon input into the TCA cycle derived from glucose and pyruvate is downregulated. A significant portion of glucose is diverted to the pentose phosphate pathway (PPP), where it is converted to ribose sugars and NADPH-reducing equivalents. Reactive oxygen species (ROS) that are increased in TRAP1 KO cells may be countered by this procedure. After decarboxylation, pyruvate typically enters the TCA cycle and aids in the creation of acetyl-CoA, an essential TCA cycle intermediate [23, 29]

A large amount of TRAP1 inhibits the respiratory complex II and leads to the generation of elevated levels of succinate, resulting in the reduction of SDH activity. Moreover, TRAP1 controls the opening of PTP in mitochondria by modulating the functions of OXPHOS complexes II and IV, which correspond to SDH and cytochrome c oxidase, respectively [30, 51]. The implications of the chaperon's capacity to transition between a dimer and a tetramer structure are currently unclear. Phosphorylation-induced activation of TRAP1 leads to downstream effects on Src tyrosine kinase and Ras/MEK/ERK signaling, enhancing its activity. Consequently, TRAP1-mediated inhibition of SDH causes an accumulation of succinate, which in turn triggers epigenetic changes, migration, and angiogenesis, as depicted in (Fig. 4). Additionally, TRAP1 promotes cellular invasion and migration through the STAT3/MMP2 pathway [29, 38, 71].

### Dysfunction of SDH

In the early 1920s, it was suggested that tumor cells could modify their glucose metabolism, resulting in a shift in their energy production towards glycolysis. This metabolic adaptation, commonly referred to as “aerobic glycolysis,” involves the generation of energy primarily through glycolytic pathways. While this hypothesis initially seems illogical, it has now been proven to be true [63]. The most efficient biological adaptation of cellular energy to facilitate rapid cancer cell growth was the reprogramming of the TCA cycle. This metabolic adjustment plays a crucial role in the development and progression of cancer through multiple stages [18, 63, 69, 81].

#### Epigenetic alteration

Consistent with topical investigations, succinate is a new “epigenetic hacker” that disrupts the process of DNA and histone demethylation [26]. Furthermore, succinylation is a post-translational alteration where a succinyl group is attached to a lysine residue through an amide bond. This alteration is believed to cause a significant change in the conformation of proteins, resulting in the masking of the positive charge on lysine. Various subcellular compartments, particularly mitochondria, undergo an overall increase in lysine succinylation due to the accumulation of succinate caused by the depletion of succinate dehydrogenase (SDH). This succinate buildup ultimately leads to an epigenetic alteration in carcinomas [57]. Studies have confirmed the role of succinate in cellular transformation and cancer at both the biochemical and genomic levels [66, 76].

#### Neovascularization

**Fig. 5:**
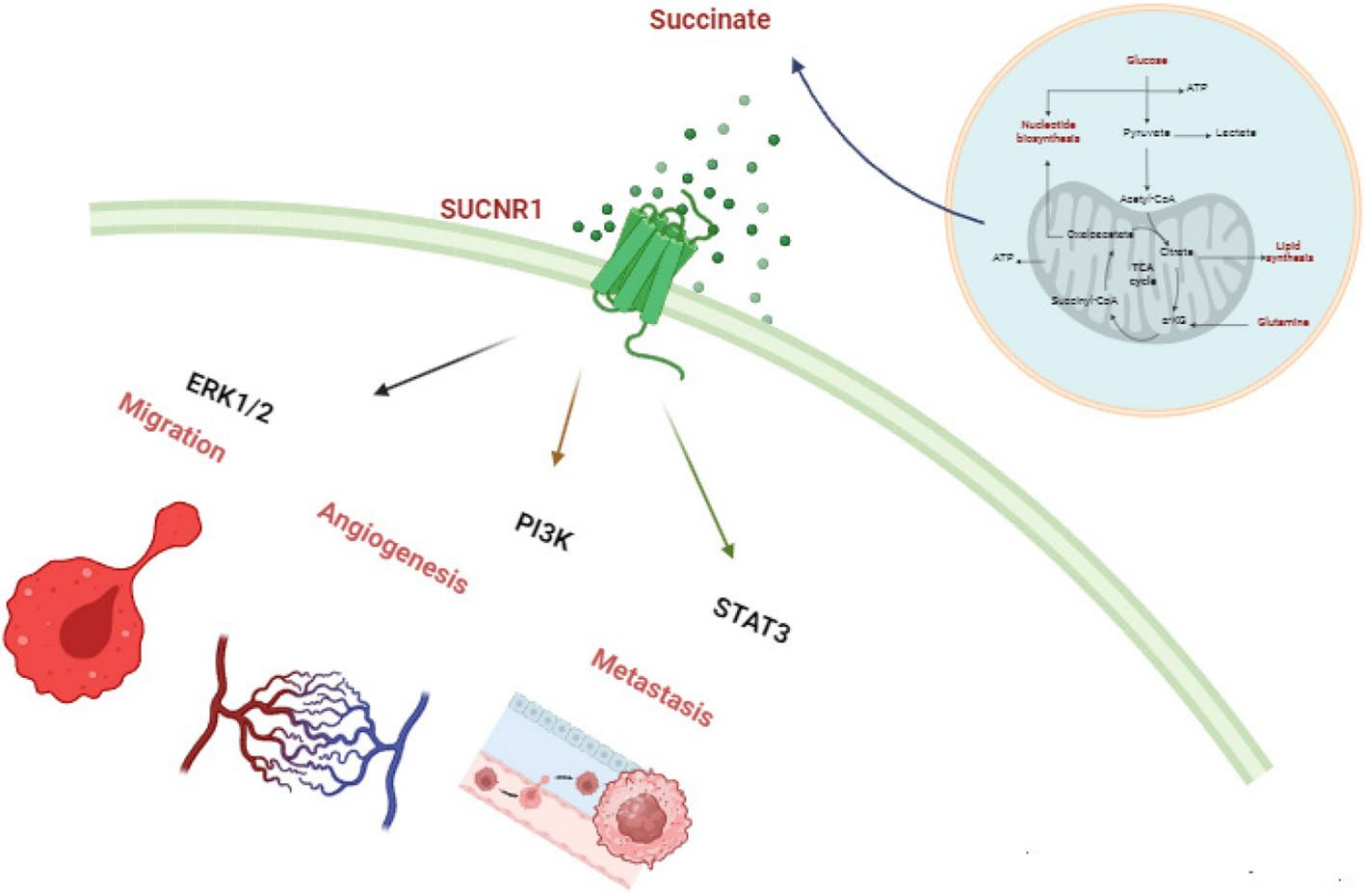
Diagram showing the signaling pathway that succinate uses to trigger cancer. The signaling mechanism that causes mitochondrial fission and succinate phosphorylation of Drp-1. ROS fuels the migration of cancer cells. Angiogenesis and metastasis are induced by extracellular regulated kinases (ERK1/2), phosphatidyl kinases (PI3K), and STAT3

Moreover, studies have shown that succinate induces cellular migration by activating the SUCNR1 receptor. This receptor also referred to as GPR91, is a member of the P2Y purinoreceptor family, which consists of G protein-coupled receptors [14]. Various tissues, such as blood cells, adipose tissue, the liver, retina, and kidney, exhibit the expression of this receptor. Recently, it has been discovered that this receptor, along with its ligand succinate, plays a crucial role in these tissues as a new regulator of local stress conditions like ischemia, hypoxia, toxicity, and hyperglycemia. Through the activation of PKC phosphorylation and subsequent activation of p38 MAPK, SUCNR1 induces cell migration as demonstrated in (Fig. 5) [39]. Furthermore, succinate triggers the formation of new blood vessels by activating extracellular regulated kinase (ERK) 1/2 and signal transducer and activator of transcription 3 (STAT3) through the specific succinate receptor GPR91. This finding indicates that this process takes place independently of the hypoxia-inducible factor (HIF) [8, 39]. A growing body of studies links the ERK1/2 signaling pathway to angiogenesis [45, 64, 75]. Furthermore, as stated by Loriot, Burnichon et al. [27], the accumulation of succinate leads to an excessive methylation process, which is capable of initiating the Epithelial-mesenchymal transition (EMT). Furthermore, Wang et al. conducted a study revealing that the depletion of SDHB results in the activation of the TGFß signaling pathway within colorectal cancer cells. This activation occurs through the enhancement of the tight-junction transcriptional repression complex SNAIL1-SMAD3/SMAD4, ultimately leading to EMT, as well as promoting cell migration and invasion [34].

### Naringenin as SDH targeting therapy

Lately, there has been a growing interest in the use of natural compounds that can hinder tumor-specific alterations in mitochondrial metabolism. These substances have the potential to serve as an appealing therapeutic approach for triggering the activation of the cellular death process in cancer cells [25, 44, 49]. Flavonoids are commonly found in fruits, vegetables, and a variety of herbal preparations. They are prevalent polyphenols in the human diet. Numerous flavonoids have demonstrated antibacterial, antioxidant, and anticancer properties [24].

**Fig. 6:**
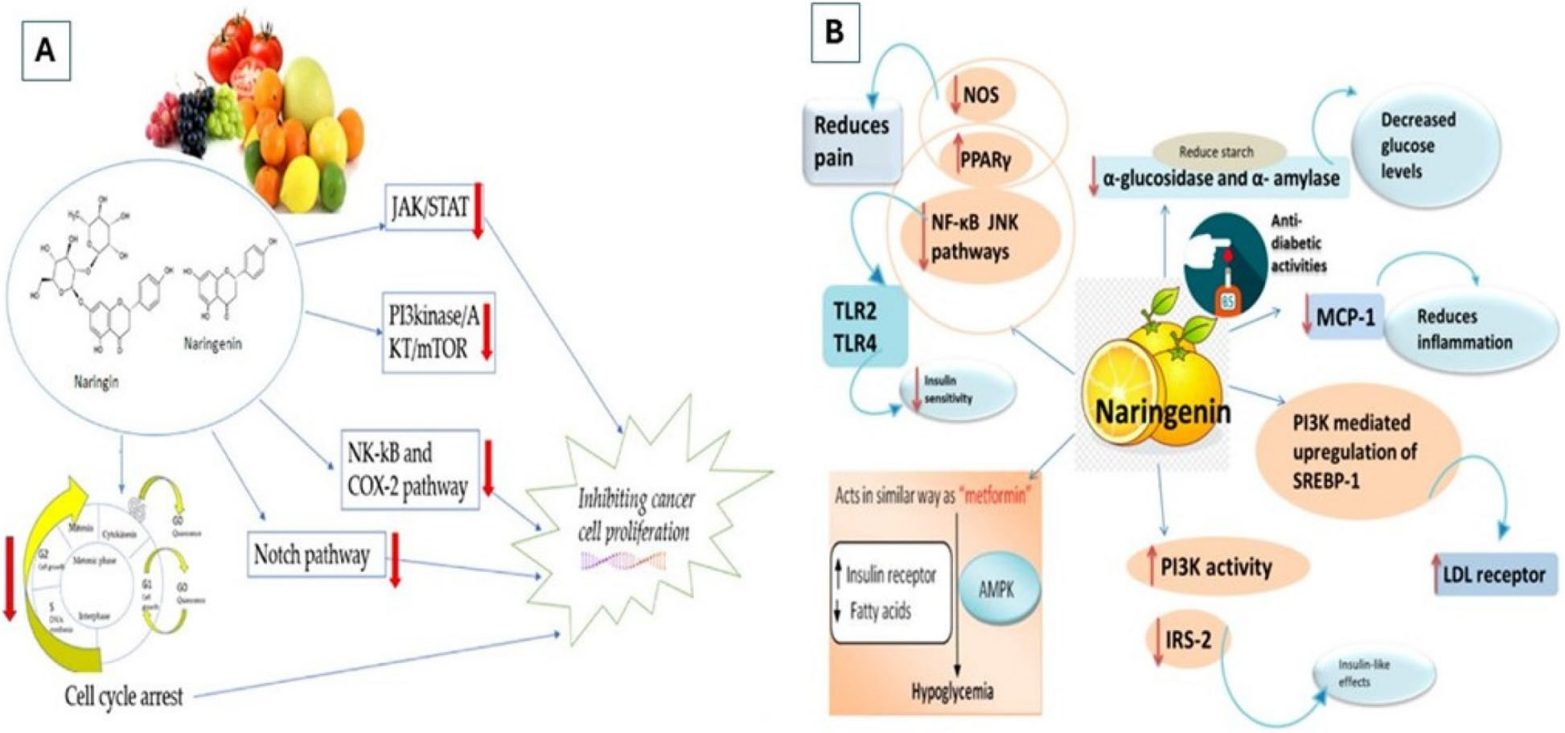
**A** Naringenin's mechanisms within the proliferation pathway. JAK: Janus kinase, a group of intracellular, non-receptor tyrosine kinases that use the JAK-STAT pathway to translate cytokine-mediated signals: transcription factors STAT, PI3K, AKT, protein kinase B, an important cell survival mediator, and mTOR, the mammalian target of rapamycin: NF-κB, also known as nuclear factor-kappa-light-chain-enhancer of activated B cells, COX-2, or cyclooxygenase 2, and Notch, a vastly conserved cell signaling system utilized by most living things [58]. **B** Several routes are implicated in metabolic syndrome control by naringenin, as well as several mechanisms underlying metabolic illnesses [1]

In 1907, Power and Tutin discovered naringenin (NAR), a member of the flavanone group (2,3-dihydro-5,7-dihydroxy-2-(4-hydroxyphenyl)-4H-1-benzopyran-4-one) [6]. NAR (C15 H12 O5) is a hydrophobic molecule with a molecular weight of 272.25 g/mol. It is mainly found as aglycones and is derived from the hydrolysis of naringin or narirutin. The primary sources of NAR include grapefruits, oranges, tomatoes, and lemons [1, 6]. Several studies have demonstrated the substantial pharmacological potential of NAR and its derivatives. These characteristics include cell cycle arrest, inactivation of carcinogens, and estrogen-like action. Furthermore, apoptosis is induced by p53 and members of the mitogen-activated protein kinase (MAPK) family, which are overexpressed and crucial in exerting pro-apoptotic effects in many malignancies (Fig. 6) [13, 65].

#### Naringenin drawbacks

However, water solubility, low oral bioavailability, and instability of NAR are mostly caused by its hydrophobic and crystalline structure. Moreover, susceptibility to rapid degradation in an acidic environment, and no specific targeting. These drawbacks provide obstacles to its effective medicinal application [24].

#### Nanodrug-drug carrier as an emerging trend in medicine

These physicochemical issues have been addressed by the development of drugs using nano-drug delivery techniques [13, 24, 47]. The surface modification of nanocarriers is primarily responsible for protecting the enclosed molecule from degradation and pH fluctuations, improving its ability to dissolve in liquids, and facilitating a controlled release of medication specifically to targeted cells. As a result, diverse types of nanocarriers have been created, including chitosan-based polymeric nanoparticles, owing to these influencing factors [54]. The progress in cancer treatment has specifically highlighted the utilization of biodegradable polymer nanoparticles such as chitosan (CS). CS consists of two subunits, D-glucosamine and N-acetyl-D-glucosamine, which are connected by a (1,4) glycosidic bond [65, 70]. the amine group found on the glucosamine unit of CS is an essential part due to its potent and responsive positive charge. This positive charge enables CS to form complexes by combining with an anion molecule. Moreover, CS can enhance the transportation of medication through cell membranes [17, 42]. Chitosan nanoparticles containing naringenin were produced by ionically crosslinking CS (positive) and trisodium polyphosphate (TPP). The surface charge of NPs is typically one of the most significant elements affecting their stability throughout the short- and long term. Zeta potential indicated that the particles' surface charge had grown in our previous study. The increase in surface charge could be due to less TPP crosslinker neutralization of the NH3 groups. Particles with a high positive charge exhibit cohesiveness, tissue permeability, and greater stability. Chitosan nanoparticles have a positive zeta potential, which makes them more stable and capable of carrying medications since they can stick to negatively charged cell membranes more readily [42]. Owing to its unique sub-cellular size relative to the microscopic system, it is expected that the formulation of CS with NAR will result in substantial intracellular absorption [70]. As a result, the anti-cancer potential of the starting molecule, in this case NAR, can be enhanced.

Our previous studies suggest that CS capsulate flavonoid drugs make it targeted to specific sites on SDH especially at the UbQ-binding site between the SDHC and SDHD subunits in recent research, which demonstrated that they greatly inhibited SDH activity in cancer cells [42, 44, 49, 53].

## Conclusion

Cellular energy metabolism is the hallmark of almost all malignancies. Unlike healthy cells, which mostly obtain their useful energy from oxidative phosphorylation, most cancerous cells rely significantly on substrate-level phosphorylation to fulfill their energy requirements. succinate buildup caused by an imbalance in SDH activity, control, or mitochondrial chaperones. Due to its effects on angiogenesis, invasion, and cell migration, as well as the generation of epigenetic and metabolic alterations, succinate is a major factor in the development of cancer. Thus, strategies that prevent succinate from piling up are required. Flavonoids have recently been a key player in the control of SDH activity. Among these is NAR, an ingredient found naturally in a wide variety of fruits. According to scientific research, there appears to be little chance of major adverse effects developing, and compared to other chemotherapy medications, it should have a safer profile. However, given the outcomes of in vitro and in vivo models thus far, additional research is needed to understand the bioavailability and release of novel formulations for encapsulating NAR, such as chitosan nanoparticles, concerning clinical studies. Thus, naringenin-loaded chitosan nanoparticles are paving the way for the creation of novel metabolic cancer treatments.

## Data Availability

Not applicable.

## Abbreviations

OXPHOS: Oxidative phosphorylation dysregulation
SIRT3: Sirtuin 3
SDH: Succinate dehydrogenase
NAR: Naringenin
SDHA: Subunit A
SDHB: Subunit B
SDHC: Subunit C
SDHD: Subunit D
SDHAF: SDH assembly factor
TRAP1: Tumor necrosis factor receptor-associated protein 1
HCC: Hepatocellular carcinoma
CypD: Cyclophilin D
PTP: Permeability transition pore
ETC: Electron transport chain
PPP: Pentose phosphate pathway
ROS: Reactive oxygen species
PTPMT1: PTEN-like mitochondrial phosphatase-1
ERK: Extracellular regulated kinase
STAT3: Signal transducer and activator of transcription 3
EMT: Epithelial-mesenchymal transition
JAK: Janus kinase
COX-2: Cyclooxygenase 2
NF-κB: Nuclear factor-kappa-light-chain
CS: Chitosan
TPP: Tri-sodium polyphosphate
